# Evolutionary Genomics of Fast Evolving Tunicates

**DOI:** 10.1093/gbe/evu122

**Published:** 2014-07-08

**Authors:** Luisa Berná, Fernando Alvarez-Valin

**Affiliations:** ^1^Sección Biomatemática, Facultad de Ciencias, Universidad de la República, Montevideo, Uruguay; ^2^Unidad de Biología Molecular, Institut Pasteur Montevideo, Montevideo, Uruguay

**Keywords:** positive selection, genome plasticity, *Oikopleura dioica*, *Ciona*

## Abstract

Tunicates have been extensively studied because of their crucial phylogenetic location (the closest living relatives of vertebrates) and particular developmental plan. Recent genome efforts have disclosed that tunicates are also remarkable in their genome organization and molecular evolutionary patterns. Here, we review these latter aspects, comparing the similarities and specificities of two model species of the group: *Oikopleura dioica* and *Ciona intestinalis*. These species exhibit great genome plasticity and *Oikopleura* in particular has undergone a process of extreme genome reduction and compaction that can be explained in part by gene loss, but is mostly due to other mechanisms such as shortening of intergenic distances and introns, and scarcity of mobile elements. In *Ciona*, genome reorganization was less severe being more similar to the other chordates in several aspects. Rates and patterns of molecular evolution are also peculiar in tunicates, being *Ciona* about 50% faster than vertebrates and *Oikopleura* three times faster. In fact, the latter species is considered as the fastest evolving metazoan recorded so far. Two processes of increase in evolutionary rates have taken place in tunicates. One of them is more extreme, and basically restricted to genes encoding regulatory proteins (transcription regulators, chromatin remodeling proteins, and metabolic regulators), and the other one is less pronounced but affects the whole genome. Very likely adaptive evolution has played a very significant role in the first, whereas the functional and/or evolutionary causes of the second are less clear and the evidence is not conclusive. The evidences supporting the incidence of increased mutation and less efficient negative selection are presented and discussed.

## Introduction

More than 140 years ago the Russian embryologist, Alexander Kowalevsky, first recognized that tunicates (also known as urochordates) have vertebrate-like characteristics and postulated that both vertebrates and cephalochordate may have evolved from a tunicate-like ancestor during or prior to the Cambrian explosion. The significance of this discovery was immediately recognized by Darwin (1871) and became an important support for his evolutionary theory (cited in the book, Descent of Man, 1871). Today it is well established that tunicates along with cephalochordates and vertebrates comprise the phylum Chordata. These three groups share several morphological features that characterize them, such as the presence of the notochord, a dorsal neural tube, gill slits, and the endostyle (Malcolm 1962). Until few years ago, the above-mentioned phylogenetic view was widely accepted, namely that tunicates occupy the most basal position in the chordate phylogeny, a view very likely influenced by the overall morphological resemblance and apparently increased complexity exhibited by cephalochordates and vertebrates relative to tunicates (that—with exception of larvaceans—show tadpole-like morphology and vertebrate features only in the larval stage, see table 1). However, the precise phylogenetic location of tunicates inside chordates started to become a matter of controversy in more recent years since new phylogenetic inferences based on morphology or fragmentary sequence data suggested conflicting interpretations (reviewed in Swalla and Smith [2008]). Unexpectedly, contrary to this traditional view, new and more robust phylogenetic analyses of chordates using collections of genes representative of their genomes strongly supported the proposal that tunicates and not cephalochordates represent the closest living relatives of vertebrates (Bourlat et al. 2006; Delsuc et al. 2006, 2008; Dunn et al. 2008; Putnam et al. 2008).

**Table 1:** Some Relevant Biological Features of Tunicates

	Oikopleura dioica	Ciona intestinalis
Entire life cycle span	4 days at 20 °C	2–3 months
Morphology: Notochord, dorsal neural tube, and muscular tail (vertebrate-like features)	Conserved throughout their life	Loss of vertebrate features in the metamorphosis (tadpole-like morphology only in the larvae)
Reproduction	Separate sexes	Hermaphrodite
Habitat	Pelagic	Benthonic
Body size	2–4 mm	Up to 20 cm
Genome size	70 Mb	160 Mb (190 Mb *C. savignyi*)
Predicted number of genes	∼18,000	∼15,300 (12,600 *C. savignyi*)
Gene density	One gene per 3.9 kb	One gene per 10.5 kb (1/15 kb *C. savignyi*)
Genome organized in operons	27% (4,997 genes in1,761 operons)	19% (2,909 genes in 1,310 operons)
Intron size	Very small introns (peak at 47 bp).	Longer introns (peak at 300 bp and a thinner peak at 60 bp)
Atypical intron boundaries	GA/AG (12% of genes)	GC/AG (512 introns, 0.53%)
Gene loss	Specific gene loss	Massive gene loss
Hox genes loss	Hox7, Hox8, Hox9, and Hox11	Hox3, Hox5, Hox6, Hox7, and Hox8
Transposons and repetitive DNA	Rare	Abundant
Population mutation rate (*θ* = 4*N*_e_*μ*)	0.022	0.012 (0.056 *C. savignyi*)
d*N*/d*S*	0.12	0.07 (0.07 *C. savignyi*)
Molecular rate	Three times faster than vertebrates	1.5 times faster than vertebrates

As a result of the key evolutionary position that they occupy and their particular developmental plan, tunicates have been extensively studied to understand the evolutionary origin of vertebrates and the mechanisms of vertebrate development. More recently several genome sequencing efforts were carried out in the most studied ascidian species, namely those of the genus *Ciona* (*C. intestinalis* and *C. savignyi*; Dehal et al. 2002; Vinson et al. 2005) and in the larvacean *Oikopleura dioica* (Denoeud et al 2010), aiming to contribute from a genomic perspective to the understanding of these fundamental aspects of vertebrate evolution and development (table 1 summarizes relevant biological features from both species).

The availability of genome sequences from these tunicates together with the genomes of the cephalochordate *Branchiostoma floridae* (the lancelet), sea urchin, and vertebrates also allowed to perform several comparative genomic analyses that provided very important insights on the evolution and organization of chordate genomes (Sodergren et al. 2006; Putnam et al. 2008). Special mention deserves the case of *O. dioica* that after the publication of its genome has become rather popular attracting the attention of a broader audience, apart from developmental and evolutionary biologists. This genome has several unique and surprising features, showing for instance that the plasticity that a genome can attain is much greater than previously suspected. But, even if *Oikopleura* may represent an extreme case of genome plasticity and evolutionary pace, all tunicates appear to share many of the genomic features that make *Oikopleura* so particular. Recent genome-wide analyses on the rates and patterns of amino acid evolution in both *Ciona* species (Berná et al 2009; Tsagkogeorga et al. 2012) as well as in *O. dioica* (Berná et al. 2012) show that not only tunicates are fast or very fast evolvers, but also they have many peculiarities in their patterns of amino acid evolution.

In this article, we review the fundamental aspects of genome and molecular evolution of this key and fascinating group of organisms. This article is divided into two parts. The first one addresses genomic plasticity observed in tunicates, whereas the second part focusses on the unusual rates and patterns of amino acid evolution and their possible evolutionary causes and implications.

## Genome Plasticity in Tunicates

This part is divided into three sections that outline the most relevant features of the genome reorganization processes that took place in the model tunicate species. The first two sections describe the mechanisms of genome reduction in tunicates, which in *Ciona* was largely caused by considerable gene loss (see Gene Loss in *Ciona* section), but in *Oikopleura*, apart from being more pronounced, it is due to compaction (see Genome Reduction in *Oikopleura* section). The last section (Poor Synteny Conservation in Tunicates) discusses synteny conservation reduction and its possible evolutionary significance.

### Gene Loss in *Ciona*

**Fig. 1.—:**
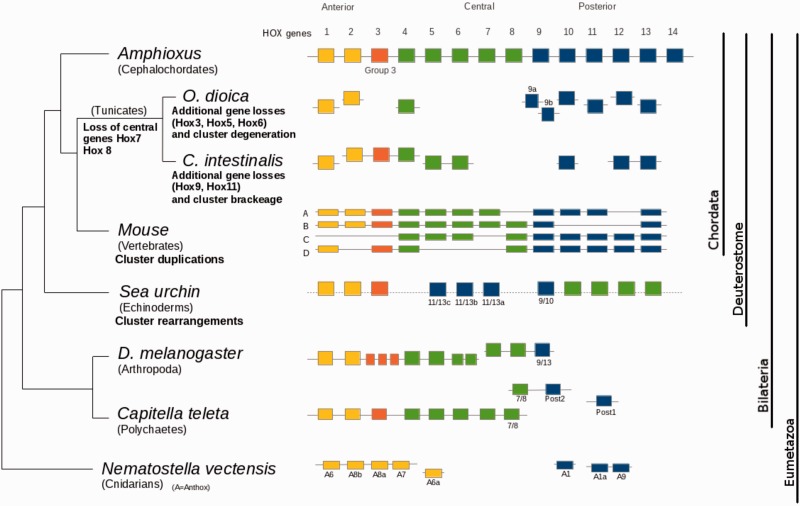
HOX gene cluster evolution. Schematic representation of the HOX cluster evolution in representative Eumetazoan groups. Hox genes are schematized as follows: anterior Hox genes (yellow), paralogy group 3 Hox genes (orange), central Hox genes (green), and posterior Hox genes (blue). Horizontal lines connecting genes indicate linkage. The lancelet cluster is considered as the canonical one, since it is complete and did not undergo rearrangements (Monteiro and Ferrier 2006). Tunicates lack some of the central Hox genes and the cluster is broken. Specifically, *Ciona intestinalis* lost four Hox genes (Hox 7, 8, 9, and 11) and the Hox cluster was separated in five syntenic segments, whereas *Oikopleura dioica* lost five Hox genes (3, 5, 6, 7, and 8) and the cluster is completely disintegrated, namely none of the genes kept synteny (Seo et al. 2004). Complex rearrangements of Hox gene order occurred in sea urchin genome (Cameron et al. 2006). However, other echinodermes present temporal and spatial collinearity for several Hox genes (Mooi and David 2008). Although some insects present a collinear Hox cluster, this is partially fragmented in *Drosophila melanogaster* (genes also referred to as: *Lab, pb, z2, zen, bcd, Dfd, Scr, ftz,* and *Antp* corresponding to the first cluster and *Ubx, abd-A,* and *Abd-B* to the second one) (Hughes and Kaufman 2002).

One of the most interesting observations concerning the *Ciona* genome is that several genes which are present in vertebrate genomes, and even in other invertebrates, appear to be absent in *C. intestinalis* (Dehal et al. 2002). This finding led to the proposal that the genome of *Ciona* has lost a significant number of genes (Holland and Gibson-Brown 2003), a suggestion corroborated by subsequent studies which showed that from 3,921 reconstructed sets of protein families present in the chordate ancestor, 798 (20.4%) were absent in the *Ciona* gene predictions (Hughes and Friedman 2005). It was also estimated that *Ciona* lost 35% and 45% more ancestral gene families than pufferfish and humans, respectively (Hughes and Friedman 2005). Some genes absent in the *Ciona* genome but present in *Drosophila* and vertebrates include those encoding for the histidine decarboxylase, the nuclear receptor tailless, the homologous to the most common circadian rhythm proteins (*Per*, *Bmal*, and *Clock*), two members of the LIM homeodomain gene family (*Lhx6* and *Lhx7*), and hemoglobins (Dehal et al. 2002). But probably the most remarkable example of gene loss is the case of Hox genes cluster (schematized in fig. 1), which normally exhibits great conservation throughout metazoans (including *Drosophila*, sea urchin, cephalochordates, and vertebrates). In *Ciona,* however, this cluster contains only nine genes and four of them (Hox7, Hox8, Hox9, and Hox11) were lost (Dehal et al. 2002). Interestingly, five Hox genes are missing in the *Oikopleura*, two of which (Hox7 and Hox8 belonging to the central group) are shared losses, thus indicating that these losses most likely took place at the base of tunicate lineage (Seo et al. 2004; Monteiro and Ferrier 2006).

The biological factors underlying this extensive lineage-specific deletion of ancestral genes and gene families are not very clear. Evidently genes could be lost if their absence is not sufficiently deleterious to prevent transmission to subsequent generations, something that depends on the functional importance of the genes in question. Hughes and Friedman (2005) suggested that these deletion events along with the acquisition of novel genes in the ascidian ancestor possibly reflect adaptations to the adult sessile life and characteristic developmental plan that these animals have. Indeed, this implies that many of the genes necessary to achieve a typical adult vertebrate-like plan would be no longer necessary. At any rate, the extent of gene loss in these organisms appears to be too ample to be associated only with changes in the developmental plan.

Finally, a cautionary note is of order since it should be taken into account the fact that the failure to find a given gene does not necessarily mean that it was effectively lost. It has been suggested that it is feasible that several of these putatively missing genes may in fact have evolved so rapidly (considering that tunicates are very fast evolvers) that today could no longer be readily recognized because they lost significant resemblance to their vertebrate orthologs (Cañestro et al. 2003). As we show in the section Causes of the Increment in Evolutionary Rates, this may have happened in some cases.

### Genome Reduction in *Oikopleura*

**Fig. 2.—:**
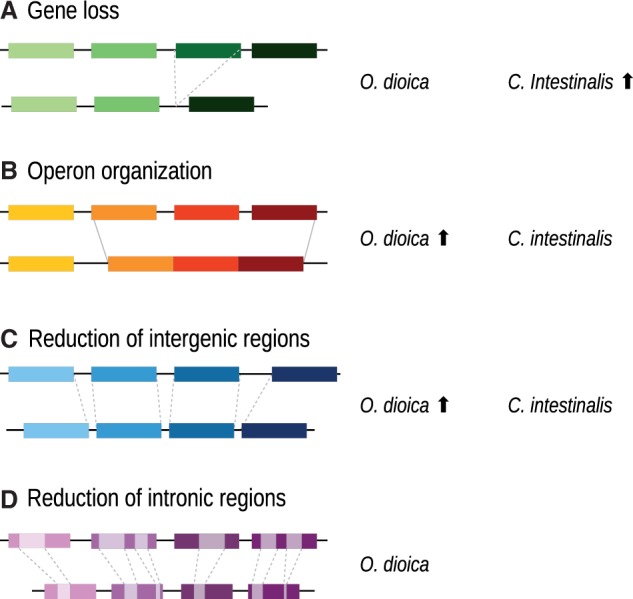
Different mechanisms of genome reduction: (*A*) Gene loss, (*B*) operon organization, (*C*) reduction of intergenic regions, and (*D*) intronic regions shrink. The arrow pointing upward next to the species name indicates that the mechanism is more pronounce in that species.

A distinctive feature of *Oikopleura* genome is that it has been trimmed on an unprecedented scale becoming extremely compact with only 70 Mb (Denoeud et al. 2010). Even if this process of genome reduction could have been caused in part by the elimination of genes (like notochord genes and Hox genes, as described in the previous section), this was not the only or even the main cause, since this genome contains about 18,000 predicted genes. Instead, genome compaction, namely packaging genes into smaller space, had a much more significant impact on genome reduction. As a result, gene density became much higher than in other chordate species. In the species from the genus *Ciona*, which also have undergone a similar, yet less extreme, process of genome compaction, gene density is between three (*C. intestinalis*) and four (*C. savignyi*) times lower than *O. dioica* (see table 1 for details on genome sizes and gene numbers). In turn, in comparison to nontunicate chordates, the difference is considerably more pronounced. Amphioxus, which is regarded as representative of the ancestral chordate, contains 22,000 genes in a 520-Mb genome (Putnam et al. 2008), whereas humans have about 25,000 genes in a genome of 3.3 Gb, meaning that they are 6 and 35 times less gene dense than *O. dioica*. This level of compaction was mostly accomplished by the three different mechanisms depicted in figure 2: Organization of genes in operons, reduction of intergenic distances, and short intronic sequences. Transposon scarcity also contributed to genome reduction.

In effect, many tunicate genes have the peculiarity of being organized in polycistronic transcription units (operons), a type of genomic organization not common in metazoans. Apart from tunicates, this type of organization has been reported only in nematodes and flatworms (Ganot et al. 2004; Guiliano and Baxter 2006). Around 1,800 operons have been identified in the *Oikopleura* genome encompassing about 27% of genes (Denoeud et al. 2010), a figure perceptively higher than that observed in both *Ciona* species where 19% are organized in this manner (Satou et al. 2008; Zeller 2010). Like in *Ciona*, the majority of operons are bicistronic (60% and 74%, respectively), but a substantial proportion contains three or more genes, whereas some have up to 11 genes (Satou et al. 2008; Denoeud et al. 2010; Ferrier 2011). Genes located in the same polycistronic transcription unit are cotranscribed and then undergo a process of maturation that besides polyadenylation involves the addition of a spliced leader in the 5′-end (*trans*-splicing) to become a mature monocistronic mRNA (for a review, see Hasting [2005]). It has been suggested that some cnidarians, like *Hydra*, might have some genes organized in operons since *trans*-splicing has been reported in this basal metazoan (Stover and Steele 2001; Chapman et al. 2010). Relevant to genome compaction, the fact that genes are organized in this manner reduces the intergenic space needed to allocate DNA segments containing transcription initiation and regulation signals. It is also important to mention, however, that *Oikopleura* has densely packed genes even outside operons, where intergenic sequences are short (<1 kb).

Another peculiarity that contributed in a very significant way to genome compaction in *O. dioica* is that the majority of genes contain very small introns. In fact, most introns are very short, being on average less than 50 nt in length, and only 2.4% of them are larger than 1 kb (Denoeud et al. 2010). Still, some genes have relatively large introns and intergenic sequences. This latter group is composed mostly by developmentally regulated transcription factors. A detailed study of *Oikopleura* introns shows an astonishing turnover rate which resulted in a massive loss of ancestral introns (Denoeud et al. 2010). Specifically, out of 5,589 mapped introns, 76% are considered as recently acquired (they occupy new positions), and only 17% were identified as old ones, being the latter in general large introns. Furthermore, the canonical splicing signal (i.e., GT/AG intron/exon boundaries) detected by the major spliceosome (the only enzymatic machinery that appears to be present in *Oikopleura*) is not observed in about 12% of introns, which have GA/AG as intron/exon boundary instead. Introns having this noncanonical intron/exon boundary are 3-fold more frequent in the population of newly acquired introns (i.e., introns that are exclusive from *Oikopleura*). Considering that these atypical introns seem to be spliced out correctly by the major spliceosome, it follows that this splicing machinery might be rather permissive, yet keeping precision. Denoeud et al. (2010) proposed that such permissiveness in conjunction with its accuracy might have favored intron gain, by its ability to accurately splice out newly acquired introns. The exact mechanisms that allow this dynamics in *Oikopleura*, namely those that lead to intron gain and loss are not fully understood. Yet, some evidences suggest that these two processes might occur through reverse transcription and recombination (Denoeud et al. 2010). Specifically, intron spread may take place in a retrotransposon-like manner using a RNA intermediate (Denoeud et al. 2010), a mechanism called reverse splicing, analogous to that used by autocatalytic group I and group II introns, whereby a free intron (that was spliced out) can be ectopically inserted into a transcript, which in turn is retrotranscribed and then reinserted back into the genome (Lambowitz and Zimmerly 2004; Roy and Gilbert 2006). Likewise, retro-insertion (at the same locus) by homologous recombination of mature (spliced) mRNA may account for intron loss.

The last worth mentioning feature that also contributed to small genome size (exclusively in *Oikopleura*) is the scarcity of transposable elements. It is worth reminding that transposons contribute considerably to genome size; for instance, in human they represent about 40% of the genome. Even compared with other compact genomes, *Oikopleura* has a much lower density of transposable elements. Besides, the majority of the so-called pan-animal transposon superfamilies (many of which are even present in *Ciona*) were lost (Denoeud et al. 2010; Chavali et al. 2011). However, *Oikopleura* contains an exclusive non-long terminal repeat (LTR) retrotransposon called Odin whose origin is not very clear, and a LTR-containing retrotransposon family called Tor, related to the Gypsy retrovirus-like elements (Volff et al. 2004). Importantly enough, sequence comparisons indicate that Tor elements are still active in *Oikopleura* since some copies exhibit about 99% sequence identity at the DNA level. In spite of being still active, these mobile elements are present in very low numbers, suggesting that their proliferation should be under strict control (Denoeud et al. 2010).

### Poor Synteny Conservation in Tunicates

Initial studies concerning the cluster of Hox genes from tunicates had already shown reduced synteny conservation in *Ciona* and complete absence in *Oikopleura*. This group of genes, with some exceptions, is relatively well conserved in bilateria (fig. 1), being the prototypical deuterostomal organization (taken as the reference) that observed in cephalochordates (Monteiro and Ferrier 2006). As schematized in figure 1, tunicates underwent not only gene losses in this cluster but also loss of linkage of its constituent genes. Although in *Ciona*, the Hox cluster was separated in five segments (located in different contigs; Dehal et al. 2002), in *Oikopleura*, it was completely disintegrated (Seo et al. 2004). Along the same line, genome-wide comparisons show that there are 17 chromosome-scale segments of conserved synteny (macrosynteny) between amphioxus and vertebrates (also called ancestral chordate linkage groups) (Putnam et al. 2008), but this conservation has been weakened in *Ciona* and almost vanished in *Oikopleura* (Denoeud et al. 2010; Irimia et al. 2012). Additionally, *Oikopleura* conservation is almost marginal for some linkage groups whose life spans in animal evolution from organisms as distant as human and *Nematostella* (sea anemones, Cnidaria) or even the sponge *Amphimedon* (Putnam et al. 2007; Srivastava et al. 2010). For example, the conservation between human chromosomal segments and *Nematostella* scaffolds is not only restricted to those that contain the Hox genes (Putnam et al. 2007), but also there are several additional cases of ancient syntenies. For example, there is a genomic block comprising 225 genes, which is linked to Hox cluster, that was already present in the eumetazoan ancestor and has retained linkage in both human and anemone genomes (Putnam et al. 2007). Furthermore, other 40 additional chromosomal segments, encompassing half of the human genome have counterparts in syntenic *Nematostella* segments (Putnam et al. 2007). By contrast, *Oikopleura* exhibits very modest levels of conservation at this chromosomal organization level. Specifically, comparing the distribution of the *Oikopleura* genes that are orthologous to those that in the aforementioned species are located in these conserved blocks allows one to perceive that they are almost randomly scrambled throughout the genome (Denoeud et al. 2010).

These observations suggest that the functional constraints that maintain gene order in metazoans would be actually very relaxed in *O. dioica* (Chavali et al. 2011), alternatively they may be indicative that this type of constraints do not exist at all in any metazoan. In effect, if *Oikopleura* can cope with these changes, what functional/evolutionary constraints precluded similar changes in other metazoans? Given enough time, is it possible that the slower evolving metazoans will eventually achieve the same degree of gene reshuffling as that observed today in *O. dioica*? This latter alternative, namely that gene position within the genome is mostly neutral (not constrained), and consequently synteny conservation would be nothing but the result of phylogenetic inertia, has been repeatedly proposed (Srivastava et al. 2008; Koonin and Wolf 2010).

As far as local synteny conservation is concerned, Irimia et al. (2012) analyzed the occurrence of gene pairs that tend to remain associated across evolution. These authors found 595 such pairs of phylogenetically unrelated genes (i.e., not resulting from duplication) that are closely physically linked in several major bilaterian lineages (half of them already present in the nonbilaterian ancestors). Most of these correspond to genes that share *cis-*regulatory sequences, or other regulatory constraints (e.g., genomic regulatory blocks). In tunicates, by contrast, this level of synteny conservation is greatly reduced. Although in *C. intestinalis* only 54 of these conserved gene pairs were found, in *Oikopleura* the loss of local synteny conservation is even more extreme since there are just eight conserved pairs (Irimia et al 2012). This almost complete absence of conservation in the latter species is in agreement with previous observations which indicated that *Oikopleura* local gene order is virtually random*,* not significantly higher than gene association expected by chance (Denoeud et al. 2010).

## Amino Acid Evolutionary Rates and Patterns in Tunicates

The second segment of this article addresses the amino acid substitution rates in the group. The first two sections present the quantification of rate increase, both in extent (proportion of the genome affected) and in degree in *Ciona* (see *Ciona*: *C. savignyi* and *C. intestinalis* Diverged from Each Other 180 Ma) and *Oikopleura* (see *O. dioica*: A Very Fast Evolver). Sections Causes of the Increment in Evolutionary Rates and Patterns of Amino Acid Evolution, Genome-Wide Acceleration, and Relaxation of Functional/Structural Constraints are devoted to discussing the possible biological/evolutionary factors that underlie this increment in evolutionary rate. Finally, section Patterns of Amino Acid Evolution, Genome-Wide Acceleration, and Relaxation of Functional/Structural Constraints presents an evolutionary phenomenon that appears to be unique of *Oikopleura*, the lack of conservation of cysteine, an amino acid which is regarded as the most conservative.

### *Ciona*: *C. savignyi* and *C. intestinalis* Diverged from Each Other 180 Ma

**Fig. 3.—:**
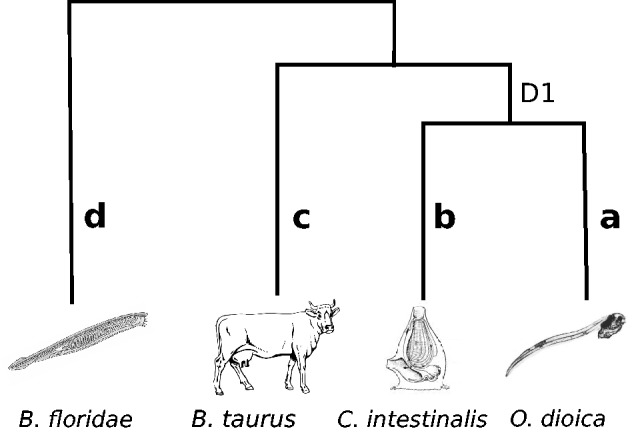
Schematic representation depicting the phylogenetic relationships of the species used to estimate the relative rate of molecular evolution on the basis of branch lengths. D1 stands for the length of the branch connecting the common ancestors of tunicates with that of tunicates and vertebrates (i.e., Olfactores); a, b, and c the lengths of the respective branches.

Early investigations on the two model species from the genus *Ciona*, namely *C. intestinalis* and *C. savignyi*, suggested that in spite of the fact of being very similar in morphology (Hoshino and Nishikawa 1985; Byrd and Lambert 2000), they seemed to be quite divergent from the genetic point of view (Johnson et al. 2004). More recently, genome-wide analyses were performed to compare distances between orthologous gene pairs from these two ascidian species with those of vertebrates (Berná et al. 2009). Specifically, genome-wide average distances and the times of divergence (as estimated from the fossil record) for nine different couples of vertebrates were used to calibrate a molecular clock and then compared these distances with distances between the two *Cionas*. Unexpectedly, the distance between the two *Ciona* species turned out to be larger than that between human and Gallus, a figure compatible with that obtained before using the huntingtin and 18S ribosomal genes (Johnson et al. 2004; Gissi et al. 2006). Such level of sequence divergence is startling if one considers that not only both species belong to the same genus and are almost indistinguishable in their morphology (there are cases of misidentification) but also that they can even produce hybrids experimentally (Byrd and Lambert 2000). In turn, this level of sequence divergence would imply that their time of separation is about 300 Myr if they had the same evolutionary pace as vertebrates. But to safely estimate the time of divergence between them using this molecular clock calibration, it is necessary to verify whether vertebrates and *Ciona* evolve at the same rate. Indeed, the substitution rate used in the molecular clock estimation is critical in assessing the timing of evolutionary events. To check this aspect, a relative rate test was conducted, which consist in comparing the speed of evolution between two species by estimating if the number of substitutions in the branches leading to each one of the stand species since their separation from their common ancestor is not significantly different (Tajima 1993). This analysis showed that for the majority of genes, *Ciona* evolves faster than all vertebrate groups, including the nonplacental mammals, which are characterized for having the fastest evolutionary rate among vertebrates (Berná et al. 2012). To quantify the degree of increment in evolutionary rates, the branch length separating *Ciona* species to their common ancestor with vertebrates (b + D1 in fig. 3) was compared with that of vertebrates to the same common ancestor (branch c). This comparison showed that on average *Ciona* species evolve 50% faster than vertebrates. Using the clock calibration mentioned above and correcting for rate disparity just described, it was possible to determine the approximate time of divergence between the two *Ciona*s in 180 Ma, a figure considerably lower than the estimation obtained without correction, still very remarkable considering that it is twice as much as the time of radiation of mammals.

### *Oikopleura dioica*: A Very Fast Evolver

**Fig. 4.—:**
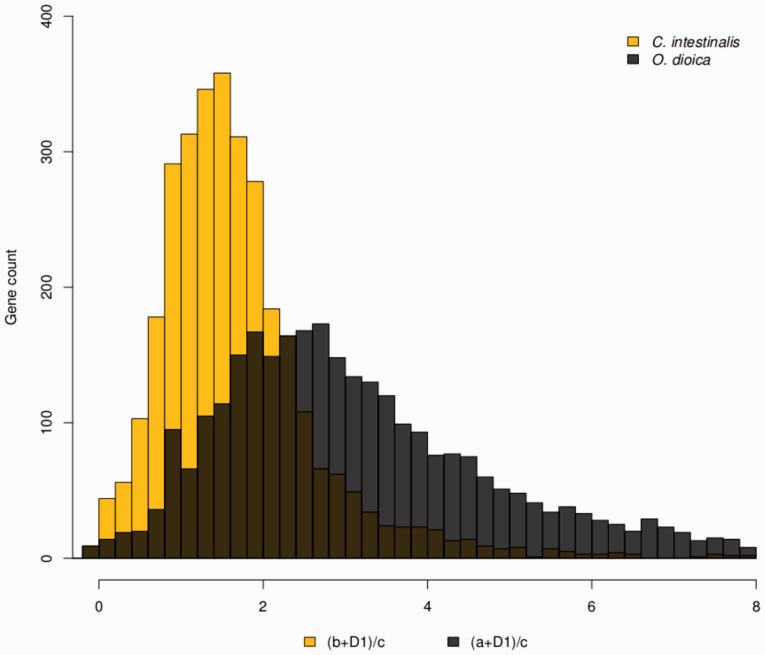
Distribution of (b + D1)/c and (a + D1)/c ratios for *Ciona intestinalis* (yellow) and *Oikopleura dioica* (dark gray). The values a, b, D1, and c correspond to branch lengths as schematized in figure 3. More specifically, a and b correspond to the branch lengths between *O. dioica* and *Ciona* to their common ancestor, respectively, and c the branch length between *Bos taurus* and its common ancestor with tunicates.

Several independent studies indicated that *O. dioica* is a very fast evolver given that this species is always represented by surprisingly long branches in phylogenetic trees of deuterostomes built using sets of concatenated genes (Delsuc et al. 2006, 2008; Putnam et al. 2008). Along the same line, Denoeud et al. (2010) conclude that *Oikopleura* is perhaps the fastest evolving metazoan recorded so far. These authors show that in most phylogenetic trees that were obtained using nine independent data sets that comprise 26 metazoan species (considered to be representative of all metazoans), *Oikopleura* presents the fastest protein evolution rate. To quantify the degree of evolutionary acceleration in *O. dioica*, both in extent and intensity, as well as to shed light on the biological factors underlying the increment, Berná et al. (2012) conducted a detailed analysis on the rates and patterns of amino acid substitutions at the genome scale level. The differences in evolutionary rates among *O. dioica*, *C. intestinalis*, and vertebrates were assessed again using the relative rate test. Regarding the comparison with *Ciona*, the average distances between *O. dioica* and either outgroup (vertebrate or cephalochordate) are much higher, almost twice as much, than those between *C. intestinalis* and the same outgroups (fig. 4*A*). This difference cannot be attributed only to a minority of genes that are very fast evolving in *O. dioica*, indeed 95% of the genes evolve faster in *O. dioica* than in *C. intestinalis*. Comparing the branch lengths that separate *O. dioica* and *C. intestinalis* from their common ancestor with vertebrates for each individual gene (fig. 4*A*), it was possible to estimate that *O. dioica* amino acid evolutionary rate is on average more than three times higher than in vertebrates (and >2 times faster than *C. intestinalis*).

### Causes of the Increment in Evolutionary Rates

Although it is well documented that all tunicates are fast evolving chordates (Denoeud et al. 2010; Tsagkogeorga et al. 2010; Berná et al. 2012), the functional/evolutionary forces that underlie the increase in evolutionary rate are not yet clear or widely accepted. It is not clear either whether the evolutionary forces responsible of such acceleration are the same for all tunicates or not. In the quest for possible answers to these questions concerning the increment in evolutionary rates in tunicates and particularly the extreme acceleration of *Oikopleura*, different plausible hypotheses have been put forward.

The first of these hypotheses relates the high evolutionary pace of tunicates with high mutation rates. The proposal, initially based on the observation that the ratio of nonsynomymous to synonymous changes is indicative of eficient negative selection (Tsagkogeorga et al. 2010), was in consonance with two features exhibited by several of these organisms. In the first place, some tunicate species, like *Oikopleura,* live in the ocean surface where they are exposed to the mutagenic effect of ultra violet (UV) radiation (Denoeud et al. 2010). We note that although this possibility holds for some tunicates like *Oikopleura*, it does not apply to others species that are benthonic yet also fast evolvers, like several ascidians (though some of them live in shallow waters like *Ciona*, where appreciable amounts of UV radiation can access). The second factor that might have increased the rate of mutation according to Denoeud et al. (2010) is the fact that many genes encoding enzymes that participate in DNA repair pathways were not found in the *Oikopleura* genome. These observations led the authors to postulate that the genes in question were presumably lost (see table 2). In our opinion, this second factor raises a number of considerations. First, it would be important to verify if these genes are also absent in the genome of *Ciona*, because, as mentioned before, the species of the genus *Ciona* are also fast evolving tunicates. Another element to be considered in relation to this proposal is that the DNA repair genes presumably absent, belong to pathways in which many of the remaining members were found in these genomes. One worth mentioning example of putatively lost gene is the case of the gene encoding the MSH3 protein, which belongs to the mismatch repair pathway. This gene was not found in *Oikopleura*, whereas other components of the same pathway such as *MSH2*, *MSH6*, *MLH1*, and *PMS1* are present (Denoeud et al. 2010). This reveals that the pathway is still active and hence indicates that the function has not been lost. Particularly significant is the presence of *MSH2*, which is the crucial enzyme because it forms heterodimers with both MSH3 and MSH6 proteins, and these two types of heterodimers have somewhat overlapping functions (both of them correct mismatches). This in turn would imply either that the *MSH3* gene is also present or the remaining proteins complement its absence. A different situation is that observed in one of the pathways coping with double-strand break repair; specifically in the nonhomologous end-joining (NHEJ) mechanisms, where none of its several component genes were detected in *Oikopleura* (Denoeud et al. 2010). Even though this is a solid indication that this reparation pathway was most likely lost in *Oikopleura*, the lack of these proteins may be related to increased genome plasticity but not to the acceleration of single-nucleotide substitutions. The last worth considering aspect concerning this proposal is related to the fact that in these fast evolving species it is more difficult to identify homologous. Specifically, as Denoeud et al. (2010) pointed out, it is likely that many of the putatively missing genes are still present in *Oikopleura* genome but it is not possible, or easy, to detect them owing to the fact that these genes have evolved so rapidly that they lost obvious resemblance to their vertebrate orthologs. Although there is no strong support for the proposal that repair genes were lost, and the proponents were very cautious in relation to it, the explanation has been considered as the most likely and very little additional work has been conducted to confirm the initial observation or explore alternative hypothesis. In our opinion, this is still an open question that requires additional assessment. Considering that new assemblies and more accurate annotation information from tunicate genomes are now available, we decided to investigate this point further. For this purpose, we conducted an exhaustive search of the missing genes in the genomic sequences of both *Ciona* and *Oikopleura*. The results, presented in table 2, give support to the notion that some of these genes are still present in tunicate genomes. Although we could confirm the absence of most of them in *Oikopleura*, for the case of *Ciona*, all but three of them were found. Moreover, as it can be observed in this table, many of these genes exhibit especially high evolutionary rates, since their orthologs among amphioxus, sea urchin, and human display levels of amino acid identity around 40%. In summary, although all the considerations just described do not rule out the mutationist hypothesis, they do indicate that the factors invoked to explain its biological basis are not fully consistent and require revision.

**Table 2:** Genes Involved in DNA Repair Pathways Putatively Absent in *Oikopleura* Genome

Mammalian DNA Repair Proteins Hypothetically Absent in *Oikopleura dioica*			*Homo sapiens*		*Sea urchin*		*Amphioxus*		*Ciona intestinalis*		*Ciona savignyi*		*Oikopleura dioica*	
			ID	Length	Identity (%)	Aln Length	Identity (%)	Aln Length	Identity (%)	Aln Length	Identity (%)	Aln Length	Identity (%)	Aln Length
DNA synthesis	DNA polymerase	*POLB*	CAG46601.1	335	64.5	335	55.1	236	63.0	335	–	–	20.8	130
Base excision repair (BER)	AP endonucleases	*APEX2*	AAH02959.1	518	37.4	599	45.9	525	–	–	38.39	534	–	–
	Short patch	*LIG3*	NP_002302.2	949	60.0	758	65.6	747	57.3	752	57.43	754	–	–
Signaling of DNA damage	Signaling of double strain break (DSB)	*ATM*	NP_000042.3	3,056	38.5	2,156	31.9	2,291	62.1	211	62.05	361	–	–
		*CHEK2*	NP_009125.1	543	53.8	435	54.6	330	39.7	489	40.85	426	–	–
Double strand break repair (DSBR)	MRN complex	*NBN*	NP_002476.2	754	38.0	332	41.5	359	–	–	–	–	30.1	113
	Homologous recombination (HR) factor	*RAD52*	NP_602296.2	418	59.3	199	47.4	213	–	–	–	–	–	–
	NHEJ	*XRXX5*	NP_066964.1	732	41.3	743	42.2	709	36.2	522	44.14	145	–	–
		*XRCC6*	NP_001460.1	609	48.7	392	47.7	579	34.9	579	42.74	241	–	–
		*LIG4*	NP_001091738.1	911	50.1	903	53.6	911	44.2	763	45.07	761	–	–
		*XRCC4*	NP_003392.1	336	25.6	125	30.4	257	22.9	205	–	–	–	–
		*NHEJ1*	NP_079058.1	299	30.1	216	31.4	220	25.3	190	–	–	–	–
		*DNA-PKc*	NP_001075109.1	4,097	41.7	2,002	39.8	1,524	39.4	660	38.93	709	–	–
		*DCLRE1C*	NP_001029027.1	692	50.0	400	48.0	390	42.7	363	–	–	–	–
	Single-stranded DNA repair	*APTX*	NP_001182179.1	302	56.0	184	58.9	175	48.5	198	51.45	173	–	–
Mismatch repair (MMR)		*MSH3*	AAB47281.1	1,137	50.3	915	39.6	507	–	–	–	–	–	–

Note.—Identity (%) stands for amino acid identity between the gene in question and its human ortholog. Aln Length refers to the size of the biggest segment that could be aligned to the human gene. The search for genes was done using HMMER 3.1 (http://hmmer.org, last accessed June 27, 2014) as described in supplementary file, Supplementary Material online. Gene identifiers for all species are available in supplementary table S1, Supplementary Material online.

**Fig. 5.—:**
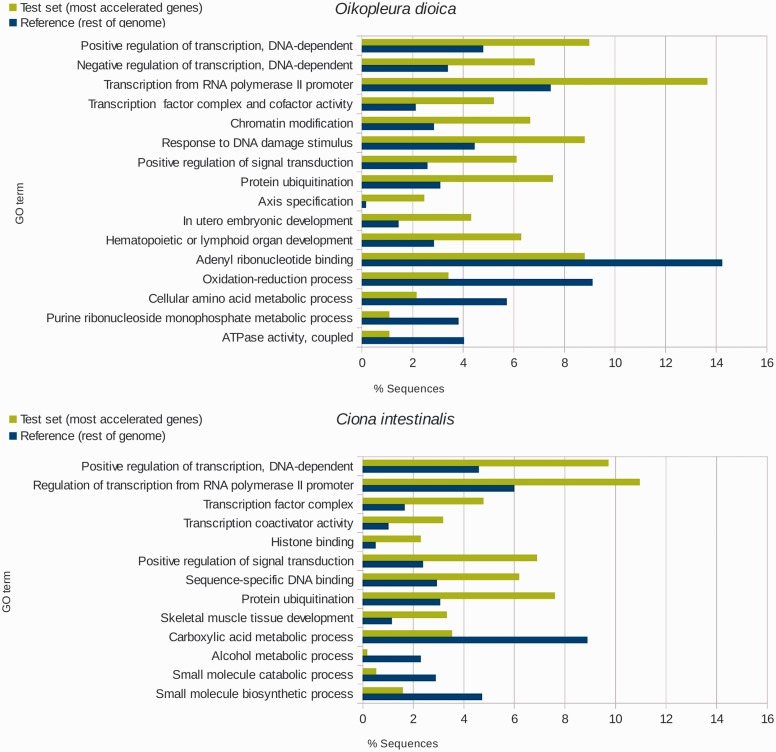
Gene ontology (GO) enrichment analysis of highly accelerated genes. Representative GO terms that exhibit statistically significant differences are shown in the graphic (Fisher’s exact test, FDR; *P* < 0.05). Distribution of GO terms for the 300 most accelerated ((a + D1)/c >5) genes from *Oikopleura dioica* (*A*) and ((b + D1)/c >2.5) *Ciona intestinalis* (*B*) and the respective reference sets (remainder of the genome). The values a, b, D1, and c correspond to the branch lengths for *O. dioica*, *Ciona*, and *Bos taurus* to the respective common ancestors, as depicted in figure 3. Modified from Berná et al. (2012).

The second group of hypotheses to explain the high evolutionary rates is related to selection, either positive, negative, or both. We conducted genome-wide analysis on the rates and patterns of amino acid evolution in *Oikopleura* and *Ciona* species and found that the most and least accelerated genes (acceleration defined as the relative increase in branch length compared with vertebrates, namely the ratios between a + D1 and b + D1 to c) are the same genes in both species, namely orthologs, in a very significant proportion (42% and 48%, respectively; Berná et al. 2012). It is important to stress that a gene with great acceleration does not necessarily have a very high evolutionary rate. In fact the group of very accelerated *Oikopleura* and *Ciona* genes have evolutionary rates that range from low to high (supplementary figs. S1–S3, Supplementary Material online). Instead, having great acceleration means that the gene in question exhibits a departing rate behavior when compared with its orthologs in other chordates (vertebrates and cephalochordates) as well as with the remaining genes from its own genome. Such departure can be either the result of chance or indicative that the gene is under a lineage- and gene-specific pressure that increases its rate. A gene ontology enrichment analysis was performed aiming to determine whether the most accelerated genes are simply a random collection or belong to some particular functional categories. The results of this analysis, presented in figure 5, show that for both species there is a clear trend in the group of genes exhibiting highest rate increment, which is very significantly enriched in regulatory proteins (spanning from transcription regulators and proteins involved in chromatin structure modification and organization to protein and metabolism regulators), development regulators (axis specification, lymphoid development, and skeletal muscle development), and poor in enzymes from the general metabolism, transporters, etc., whereas the least accelerated genes present a completely different trend (supplementary fig. S4, Supplementary Material online). In other words, this result suggests that although the process of acceleration affects all genes throughout the genome, it is particularly intensified in genes related with a broad variety of regulatory processes.

An important conclusion that can be drawn from these two observations (partially overlapping groups of very accelerated genes between *Ciona* and *Oikopleura* and similar type of genes affected in both species) is that the processes of acceleration in the genomes of both *Ciona* and *Oikopleura* are affected in a similar qualitative way, and possibly driven to some extent, by similar evolutionary or functional forces. The second important corollary that emerges from these observations is that increased mutation rate is not a likely cause of the extreme acceleration exhibited by this important group of genes. This conclusion is grounded on the expected signature of this evolutionary force, since an increment in the rate of mutation would affect the whole genome to a similar extent. Actually, there is no obvious reason why higher mutability would principally affect (i.e., produce higher acceleration) genes encoding for particular type of functions as observed here. These observations, on the contrary, point to selection, either less intense purifying (negative) selection or most likely adaptive evolution (i.e., positive selection) as the most probable causes of the extreme acceleration. We consider the latter as a better explanation because the former is more often associated, like increased mutation rate, with genome-wide phenomena (e.g., when there is a reduction in population size). Although it could also be related with groups of genes whose functions have been largely relaxed; for instance, an environmental change may render a formerly useful gene worthless, leading to a weakening on the negative selective pressure. However, if such was the case, it is also expected that this will be accompanied by pseudogenization and rapid gene loss (a nice example is that of the umami taste receptor gene from the giant panda; Zhao et al. 2010). On the other hand, if adaptive evolution was the factor underlying this unusual acceleration, then it is to be expected that it would affect the restricted group of genes encoding proteins whose functions have changed, or proteins that help the organism to get adapted to new or changing environments.

Solid evidence has been recently presented indicating that positive selection is an important, and perhaps pervasive, factor in tunicate protein evolution. Tsagkogeorga et al. (2012) using RNAseq data analyzed the interspecific variation (fixed changes or substitutions) between two cryptic *Ciona intestinalis* species termed A and B (Caputi et al. 2007) versus the intraspecific variability inside *C. intestinalis* B (Tsagkogeorga et al. 2012). The comparison was based in the well-known McDonald–Kreitam test, which basically consists in contrasting the ratio of nonsynonymous to synonymous substitutions (d*N*/d*S*) versus the ratio of nonsynonymous to synonymous intraspecific variation (p*N*/p*S*) (McDonald and Kreitman 1991). The rationale for this comparison is based on the expectation that advantageous amino acid changes (i.e., positively selected) will not last for long as polymorphisms because they will be rapidly driven to fixation by selection. As a consequence, the ratio p*N*/p*S* becomes lower than d*N*/d*S* ratio. The figures obtained for *C. intestinalis* indicate that a large fraction (between 50% and 78%) of amino acid changes would be under positive selection, a figure that is even larger than that observed in other species like *Drosophila* (Smith and Eyre-Walker 2002). Regretfully, these authors did not check whether the genes that according to this criterion contain more positively selected sites correspond to regulatory proteins.

### Patterns of Amino Acid Evolution, Genome-Wide Acceleration, and Relaxation of Functional/Structural Constraints

As far as the less pronounced, yet genome wide, acceleration is concerned, it is very unlikely that it could be the result of positive selection since adaptive evolution seldom produces a genome-wide phenomenon like this one involving almost all genes. On the contrary, it is to be expected that other factors that are intrinsically able to produce a simultaneous effect on the whole genome are the real forces underlying this process. The two likely candidates are less intense negative selection (due to decreased selection efficiency or to relaxation of functional constraints) and increase in the mutation rates. Regarding the latter, although the two groups of evidences discussed in the previous section (namely those related with initial explanation concerning the biological causes of increased mutability) imply some difficulties, and also that increased mutability is not the probable cause of exacerbated acceleration observed in genes encoding regulatory proteins, the mutationist proposal cannot be disregarded without further consideration. Whether reduced negative selection or elevated mutation rates are the factors behind the genome-wide acceleration can be tested using DNA sequence data since these two forces make different predictions. Basically, less efficient negative selection would result in an increase in the d*N*/d*S* ratio, provided that it would lead to higher nonsynonymous evolutionary rates but synonymous rates would remain basically unchanged because they are already largely free of constraints. This, of course, is under the assumption that silent positions are effectively neutral, an assumption that very often does not hold (see Shabalina et al. [2013] and references therein). An increase in the mutation rate, on the other hand, is not expected to produce any significant variation in the d*N*/d*S* ratio since increasing the rate at which mutation are introduced in populations will rise up both synonymous and nonsynonymous changes to a similar extent (Itoh et al. 2002). Large-scale population analysis, which consisted in sequence comparisons among *Oikopleura* populations from Eastern Pacific and Eastern Atlantic, revealed that although the d*N*/d*S* values were low enough to be consistent with strong purifying selection at the amino acid level, they are considerably higher than in *Ciona* (table 1). In turn, the value of the population mutation rate (i.e., the parameter θ = *4N*_e_*μ*), as estimated from polymorphism data, is quite large, twice as much as that observed in *C. intestinalis* (see table 1), but one half of that of *C. savignyi* (Vinson et al. 2005; Small et al. 2007). This is an indication that *Oikopleura* has big effective population size and/or high mutation rates. These two observations would indicate that the genome-wide (less extreme) acceleration is most likely the result of increased mutability rather than relaxed negative selection. Likewise, population genomic analyses in *C. intestinalis* are also compatible with strong purifying selection and high mutation rates. Specifically, Tsagkogeorga et al. (2012) analyzed the patterns of intraspecific variability and substitution rates in eight individual from *C. intestinalis* B. They found that the ratio d*N*/d*S* was very low indicating efficient negative selection at the amino acid level, whereas the extent of synonymous polymorphisms was very high and hence indicative of elevated mutation rates (and/or large effective population size). Importantly enough, the differences in the ratio of nonsynonymous to synonymous changes between *Ciona* species and *Oikopleura* would indicate that negative selection is more efficient in the former than in the latter. Finally, it is worth commenting that the enormous population mutation rate (*4N*_e_*μ)* reported for *C. savignyi* (Vinson et al. 2005; Small et al. 2007), which is the double of that of *O. dioica*, is somewhat puzzling. As noted by Vinson et al. (2005), the extreme heterozygosity figures observed in this species cannot be attributed to population admixture (that eventually would produce enormous variability) since the single-nucleotide polymorphism density distribution is geometric, as in random mating populations. This would indicate that in this species, as in *Oikopleura*, the very high heterozygosity is the result of large population size or very high mutation rate. Small et al. (2007) tested these alternative possibilities by inferring the effective population size (*N*_e_) using recombination frequencies (which were assessed by haplotype block length distribution). They estimated that the effective population number in *C. savignyi* would be about 1.5 million individuals, and the mutation rate (*μ*) ranging between 1.3 × 10^−^^8^ and 7.6 × 10^−^^9^ mutations per nucleotide. One immediate prediction of this *N*_e_ value is that negative selection would be very effective, being able to remove alleles with very small selection coefficients, resulting in a decrease of amino acid evolutionary rate. However, the *d*N/*d*S ratio is 0.07, which is almost identical to that reported for *C. intestinalis* (Tsagkogeorga et al. 2012), and the substitution rate is approximately the same as that of *C. intestinalis* (Berná et al. 2009, 2012).

In other words, these analyses appear to favor the notion that high mutation rates and not relaxation of functional constraints and/or inefficient purifying selection are responsible of elevated evolutionary speed in tunicates, although the differences between the two groups of tunicates might result in part from lower negative selection intensity in *Oikopleura*.

**Fig. 6.—:**
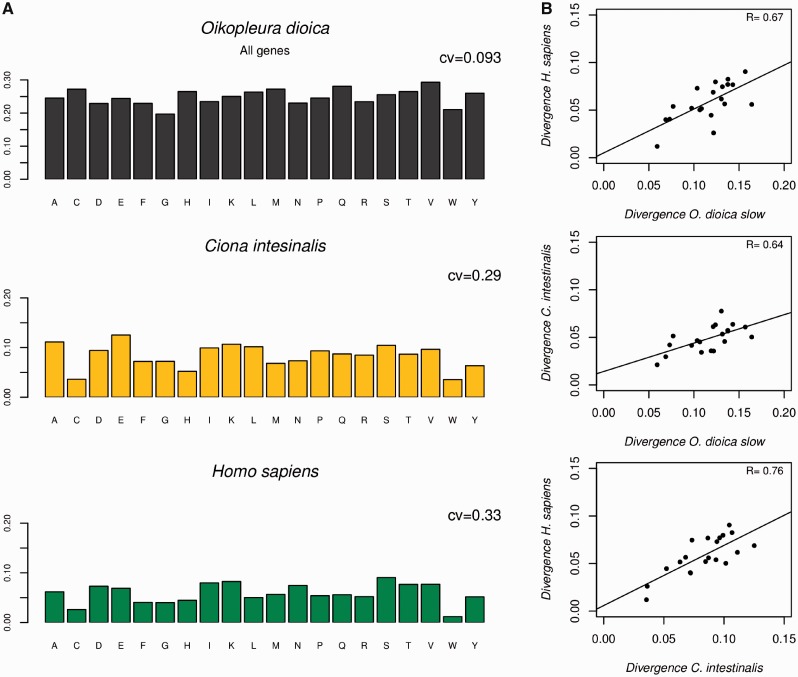
(*A*) Proportion of divergent positions refers to the fraction of nonconserved positions for each amino acid between the extant species and the ancestor of reference (for cases of *Oikopleura dioica* and *Ciona intestinalis* the ancestor would be that of tunicates). The pattern in human is shown for reference. CV stands for the coefficient of variation (i.e., variance over mean) in substitution rates. (*B*) Scatter plot of amino acid divergence patterns in the group of slow evolving genes from *Oikopleura* versus the pattern observed in *Ciona* and human. The lowest panel is a scatter plot of amino acid divergence patterns between *Ciona* and human included to illustrate the degree of similarity that “normal” divergence patterns exhibit.

In our opinion, the evidence analyzed above does not allow to definitively rule out less efficient negative selection as an important factor that differentiates tunicates from the remaining chordates, because these comparisons involve either divergence between closely related taxa (either populations or incipient species), or intrapopulation variability, hence the approach addresses only recent evolutionary events but not the long-term evolutionary history of these organisms. For this reason, this point was investigated from another perspective by examining the amino acid substitution patterns, something that can reflect more permanent evolutionary trends. These analyses consisted in determining whether there are amino acids that are evolutionary more stable than others and which amino acids are replaced by others during the course of evolution (Berná et al. 2012). The results from these analyses show that in *O. dioica* all amino acids exhibit approximately the same degree of divergence (around 25%) (fig. 6*A*). This uniformity in amino acid substitution rates is a symptom of relaxation of structural constraints because different amino acids have different intrinsic evolutionary rates; something associated with the structural roles they play (Fiser et al. 1996). For instance, the protein surface, which is occupied mostly by hydrophilic residues such as E, K, and N, is under less stringent constraints and thus tends to evolve faster than the protein core whose residues are under higher structural constraints and has a marked preponderance for hydrophobic amino acids (M, V, I, L, and F). Therefore, the homogeneity of amino acid rates seems to imply that these constraints have been relaxed. This interpretation is reinforced by the fact that when one considers only slow evolving proteins (which are under higher functional constraints), there is a clear differentiation among amino acids in their degree of conservation/divergence, which in turn is coincident with the pattern observed in other chordate species (fig. 6*B*). In other words, this means that in *Oikopleura*, the increase in evolutionary rate is accompanied by increased homogeneity across amino acids in their evolutionary speed. However, such parallelism between homogeneity and rates is not observed in *C. intestinalis*. Instead, this species exhibits a substitution pattern with substantial inter amino acid heterogeneity and similar to that of vertebrates (fig. 6*A* and *B*). This observation is another indication that negative selection seems to be more efficient in *Ciona* than in *Oikopleura.* In summary, it is possible to conclude that even if present-day tunicates are very likely under strong purifying selection, which in turn is in line with their large population sizes, this condition has not necessarily been always this way considering that complementary analyses (that reflect long-term evolutionary trends) suggest that in the lineage leading to the very fast evolving *O. dioica*, negative selection was not strong enough or functional constraints have been largely relaxed.

### Unusually High Evolutionary Rate of Cysteine in *O. dioica*

Figure 6*A* also shows an unusual feature, cysteine is one of the less conservative amino acids in *Oikopleura*. The lack of conservation of this amino acid is not only observed in fast evolving proteins but also in proteins exhibiting the lowest evolutionary rates and, so to speak, more “normal” conservation pattern (Berná et al. 2012). This deserves special consideration because cysteine is a very conservative amino acid. In fact, according to classical assessments of protein evolution, cysteine is the second most conserved amino acid after tryptophan (Dayhoff 1978; Henikoff and Henikoff 1992), or even “by far” the most conservative according to more recent reassessments (Fiser and Simon 2000; Marino and Gladyshev 2011). The conservation of cysteine is related with the fundamental role of this amino acid, in protein structure stability and function. The structural role of cysteines is well known, they form stabilizing disulfide bonds. Cysteines also play another important structural function as ligand-bound coordinating metals such as iron, zinc, or copper. The replacement or deletion of cysteine could be dramatic for the overall structure or function of the protein. In cytosolic proteins, however, cysteine generally does not form disulfide bridges, and many of their very reactive thiol groups are exposed on the surface of the protein. Some of these exposed thiols participate in a cysteine-based redox sensing and signaling system discovered in the last few years. This system to sense redox changes is based on reversible covalent modification of cysteine residues by reactive oxygen species (Jones and Go 2011). Importantly enough, in most species, the evolutionary conservation of exposed cysteines is considerably lower than that of those forming disulfide bridges (Marino and Gladyshev 2011), meaning that although important, the former can afford more evolutionary plasticity.

It would be of interest to investigate the relationship between cysteine conservation and protein disorder, since as mentioned above, this amino acid plays a central role in protein stability. However, it is not obvious a priori how this relationship will be, since some of the structures where cysteine might play a stabilizing function, such as loops, are very often (but not always) intrinsically disordered (Linding et al. 2003).

In conclusion, these observations raise some pertinent questions such as: Are structural cysteines (in extracellular proteins) more conserved than “functional redox sensing” cysteines also in *Oikopleura*? Is this lack of conservation an indication that *Oikopleura* underwent a generalized process of reorganization of its redox systems to cope with the very oxidizing environments where they live? If disulfide bonds are eliminated, what kind of stabilizing forces compensate their absence? It is thus clear that more detailed analyses are needed in order to shed light on these so peculiar facets of *Oikopleura* biology.

## Conclusions

Tunicates are so interesting and unique biological models not only because of their particular developmental plan, distinctive morphology, and key evolutionary location but also due to their genome plasticity, evolutionary rates, and patterns. Here, we reviewed the latter aspects, comparing the similarities and specificities of two model species of the group: *O. dioica* and *C. intestinalis*. The former is indeed an extremely fast evolver, being on average twice as fast as the already fast evolving *C. intestinalis*. In addition, *Oikopleura* has undergone a severe process of genome reduction and compaction, a process that in part can be explained by the loss of some genes but in reality is largely due to other mechanisms such as shortening of intergenic distances, miniaturization of introns, and scarcity of mobile elements. On the other hand, the genome reorganization in *Ciona* went in a similar direction but was not nearly as extreme. The species from the genus *Ciona* did suffer some significant gene losses and reorganization of families, incremented gene density, and a significant proportion of genes are organized in operons, but retaining considerably synteny conservation and overall the pattern of genome organization is not as dissimilar when compared with other chordates (see table 1).

Two different and somewhat independent processes of increase in amino acid evolutionary rates appear to have taken place in the genome of tunicates. Although one of them is more extreme, but restricted to genes encoding some kind of functions (mostly regulatory), the second one is less pronounced and affects the whole genome. Although the biological factors underlying this extreme acceleration are not yet completely clarified, it is likely that adaptive evolution has played a significant role. On the one hand, gene ontology analysis of the coding sequences exhibiting the greatest acceleration indicates that this group is very much enriched in transcription regulators, chromatin remodeling proteins, and metabolic regulators. This finding is suggestive that positive (adaptive) selection is a likely cause, since it is to be expected that this force would not affect the whole genome but instead a restricted group of genes that help the organism get adapted to new environments or to new challenges. On the other hand, comparative analyses of interspecific variation (fixed changes or substitutions) versus intraspecific variability also point to adaptive evolution as an important force in tunicate evolution.

As far as the possible functional and/or evolutionary causes of the less extreme, yet genome-wide acceleration is concerned, the situation is not as clear and the evidence is conflicting. First, regarding one of the factors initially invoked to explain it, namely increased mutation rates resulting from the lack of key repairing enzymes, as discussed above it has weak support given that one of the most relevant pathways, that is, the one responsible of correcting mismatches, seems to be active in *Oikopleura,* and most of such gene losses did not take place in *Ciona*. Concerning the possibility that the increase in rates is due to less effective purifying selection, several factors should be considered. First, *Oikopleura* reminds a microorganism in the sense that they have huge population size and very short generation times. This would indicate that negative selection would be very effective in eliminating even mild deleterious variants. Such interpretation is in agreement with results coming from population genomics studies that combine interspecific divergence with intraspecific variability. Moreover, the very short generation time implies that the effective mutation rate per year is substantially increased in this species. These two aspects considered together favor the view that the acceleration is most likely due to higher mutability and not to less efficient purifying selection or relaxation of functional constraints. However, it should be taken into consideration that this scenario refers to present-day situation of tunicates and hence addresses only recent evolutionary events. But analyses that tackle the long-term evolutionary history of these organisms such as the pattern of amino acid substitutions suggest that these patterns are also compatible with relaxation of functional constraints, at least in *Oikopleura*, since all amino acids have exceptionally homogeneous levels of divergence. In addition, the enormous genomic plasticity in *Oikopleura* is also commonly interpreted as the result of relaxed selective constraints (Holland and Gibson-Brown 2003).

The overall picture has been described as *O. dioica* representing *“*the most extreme case of derivation characterized so far” that has traveled much farther in the same “evolutionary journey” toward the derived genome that other tunicates like *Ciona* have “traveled to a lesser degree” (Ferrier 2011). Even if this point of view is precise for the genome organization aspects as well as for the behavior displayed by regulatory proteins, it is not possible to conclude that in *Ciona*, the genome-wide increase in amino acid rates could be attributed to the same factors (relaxation of functional constraints) like in *Oikopleura*, because contrary to what happens in the latter species, all amino acids in *Ciona* exhibit “standard” divergence patterns, which in turn are very similar to those of vertebrates.

## Supplementary Material

Supplementary file, figures S1–S4, and table S1 are available at Genome Biology and Evolution online (http://www.gbe.oxfordjournals.org/).

Supplementary Data
