# Supplementary material for: Evolutionary Genomics of Fast Evolving Tunicates
Source: Genome Biol Evol. 2014 Jul 8;6(7):1724–38. doi: 10.1093/gbe/evu122 (PMC4122922; doi:10.1093/gbe/evu122)
Supplement: Supplementary Data [file supp_6_7_1724__index.html]

Evolutionary genomics of fast evolving Tunicates — Evolutionary Genomics of Fast Evolving Tunicates — Supplementary Data 

# Evolutionary Genomics of Fast Evolving Tunicates

## Supplementary Data

files

**Files in this Data Supplement:**

- Supplementary Data - pdf file
- Supplementary Data - docx file
