# Supplementary material for: Evolutionary Genomics of Fast Evolving Tunicates
Source: Genome Biol Evol. 2014 Jul 8;6(7):1724–38. doi: 10.1093/gbe/evu122 (PMC4122922; doi:10.1093/gbe/evu122)

Supplementary file 2 (not for publishing, only for editor and referees)

Figure FR1. Correlation of branch lengths

Distance between the common ancestor of tunicates to Oikopleura (branch a)

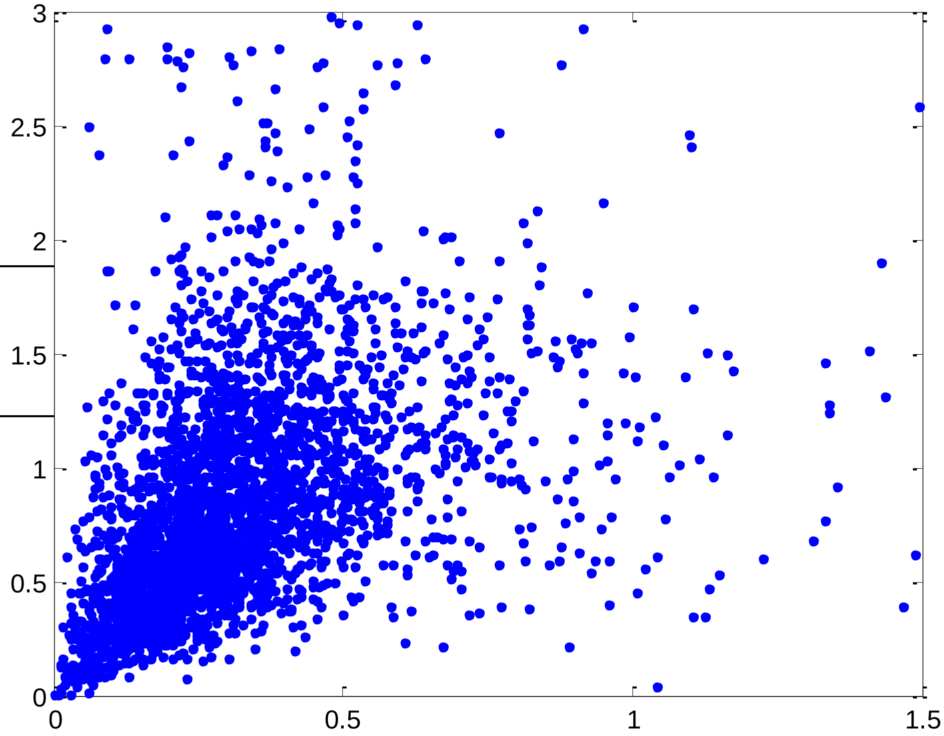

Distance between the common ancestor of tunicates with vertebrates to Bos taurus (branch c)

Distance between the common ancestor of tunicates to Oikopleura (branch ***a***)

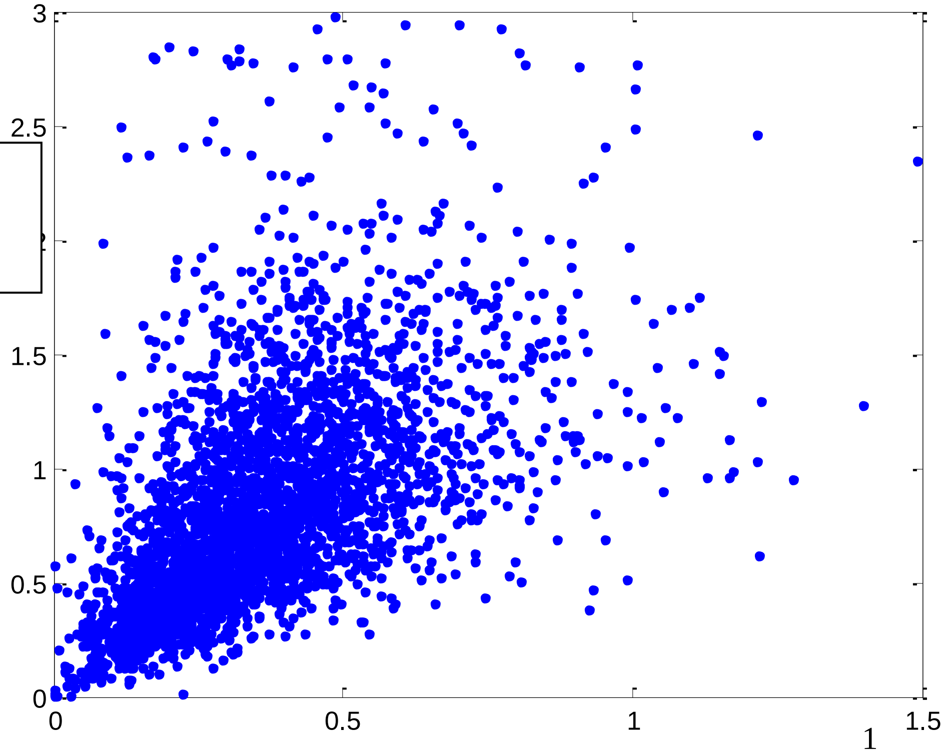

Distance between the common ancestor of tunicates to Ciona (branch **b**)

Figure FR2. GO enrichment analysis in fastest, intermediate and slowest evolving genes. Oikopleura

The fast group is composed by the 300 fastest evolving genes.

The intermediate group includes the 300 genes that go from position 1450 to 1750 in the rate rank.

The slow group the last 300 genes (the 300 slowest)

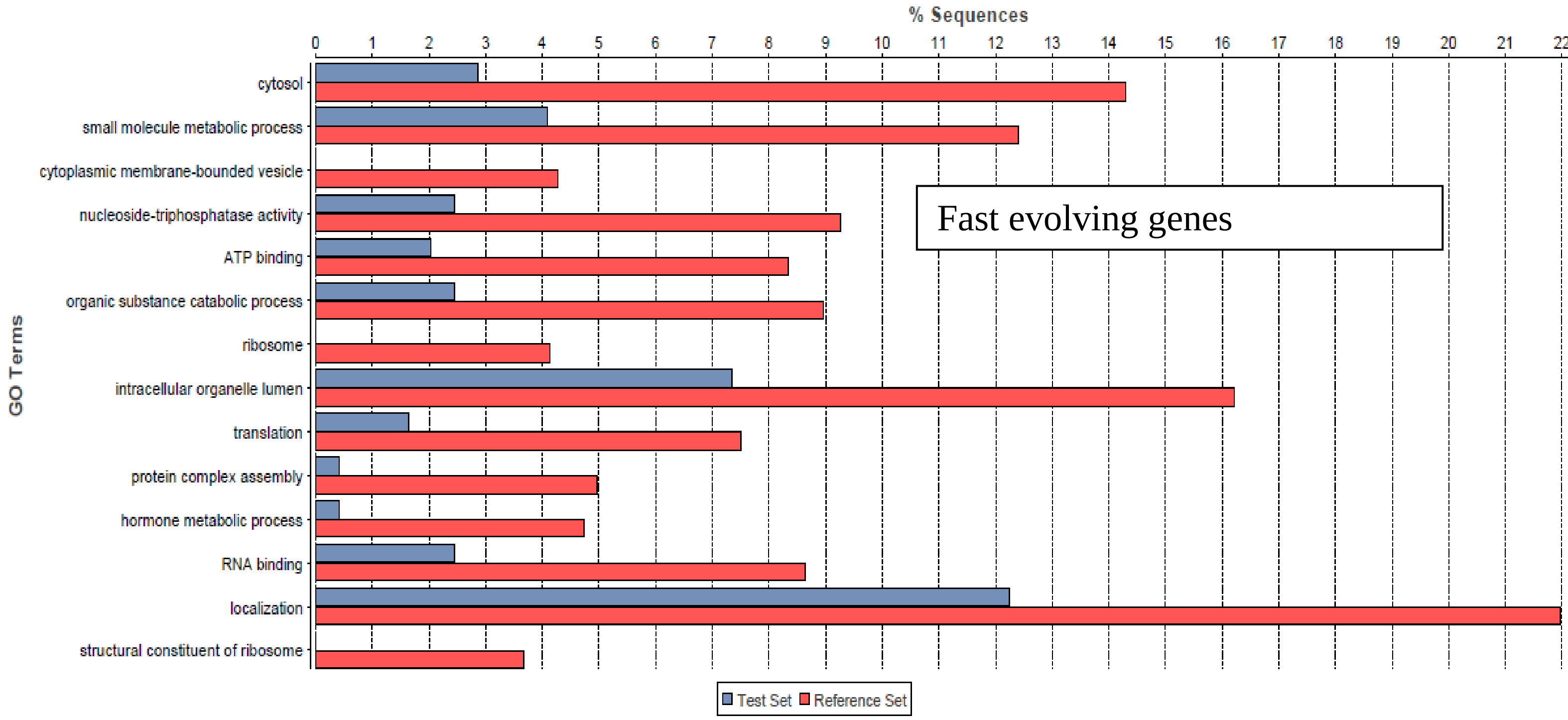

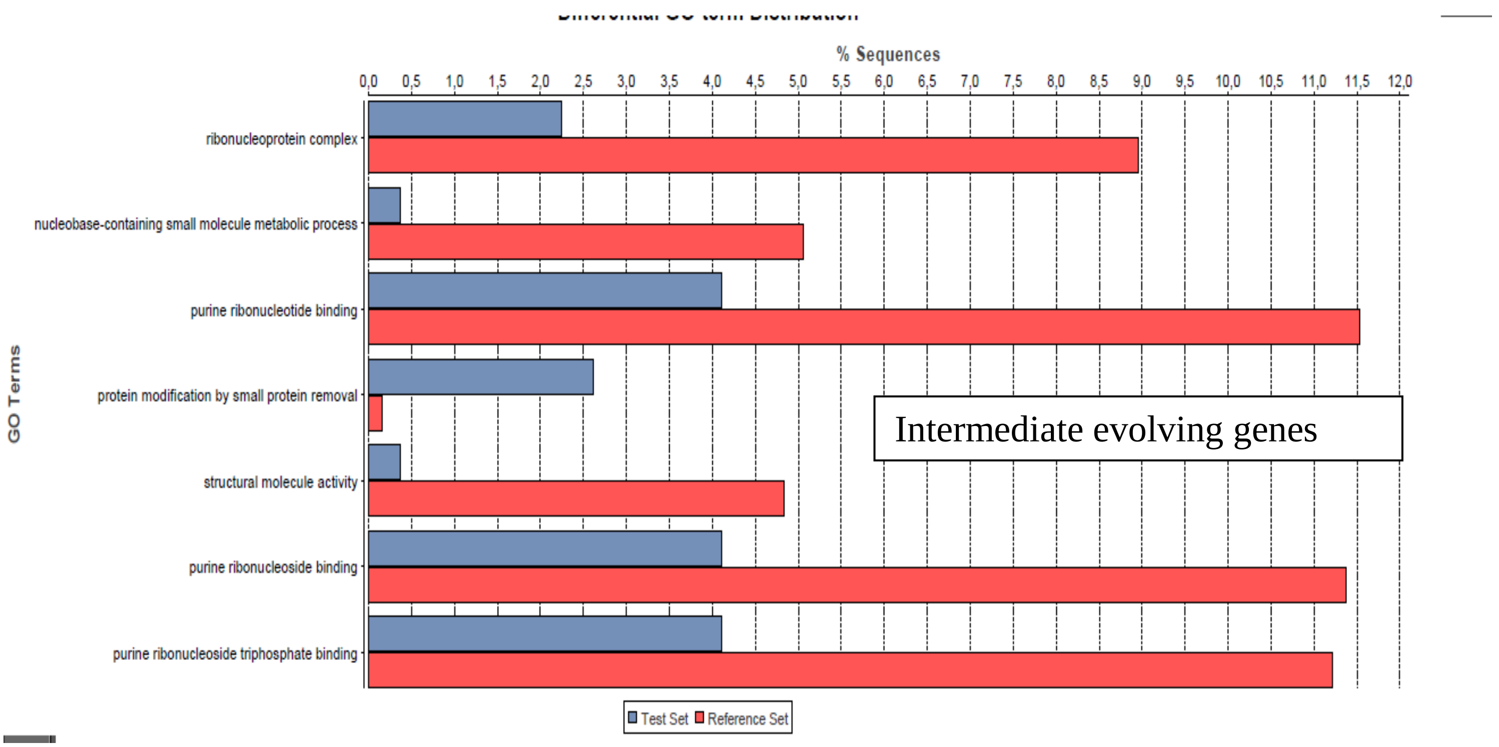

Slowest evolving genes (Oikopleura)

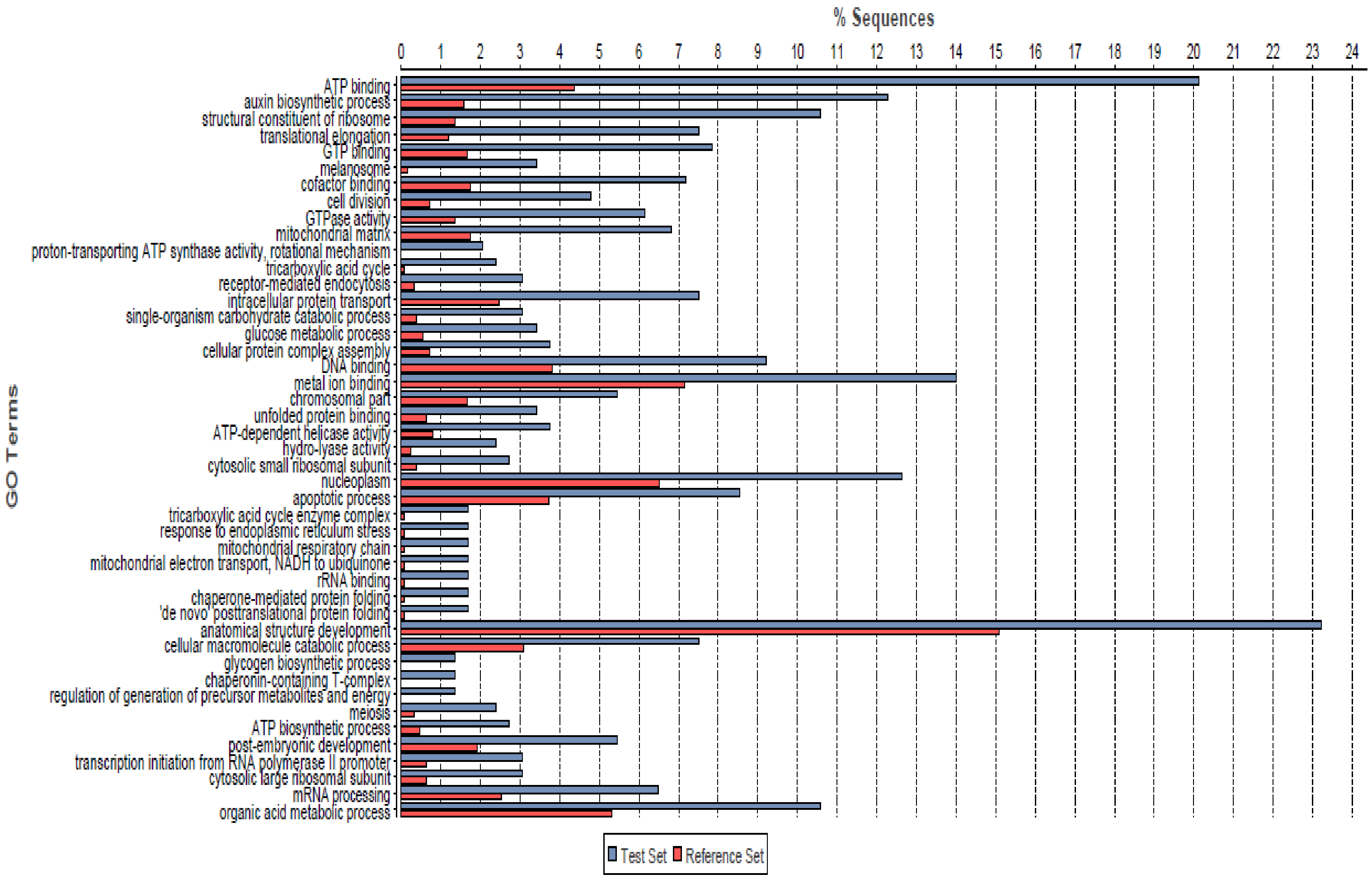

GO enrichment analysis in fastest, intermediate and slowest evolving genes. Ciona

The fast group is composed by the 300 fastest evolving genes.

The intermediate group includes the 300 genes that go from position 1450 to 1750 in the rate rank (not show because there are no significant enriched categories).

The slow group the last 300 genes (the 300 slowest)

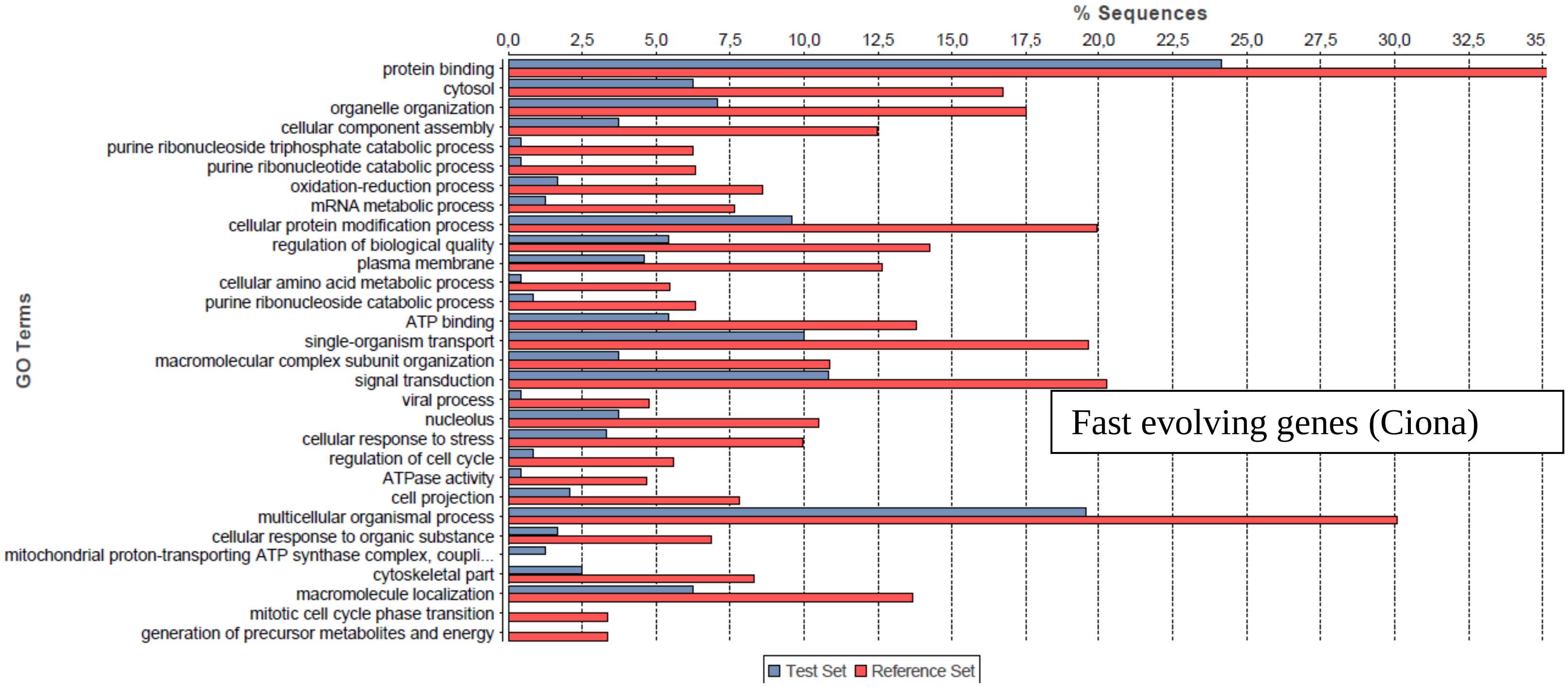

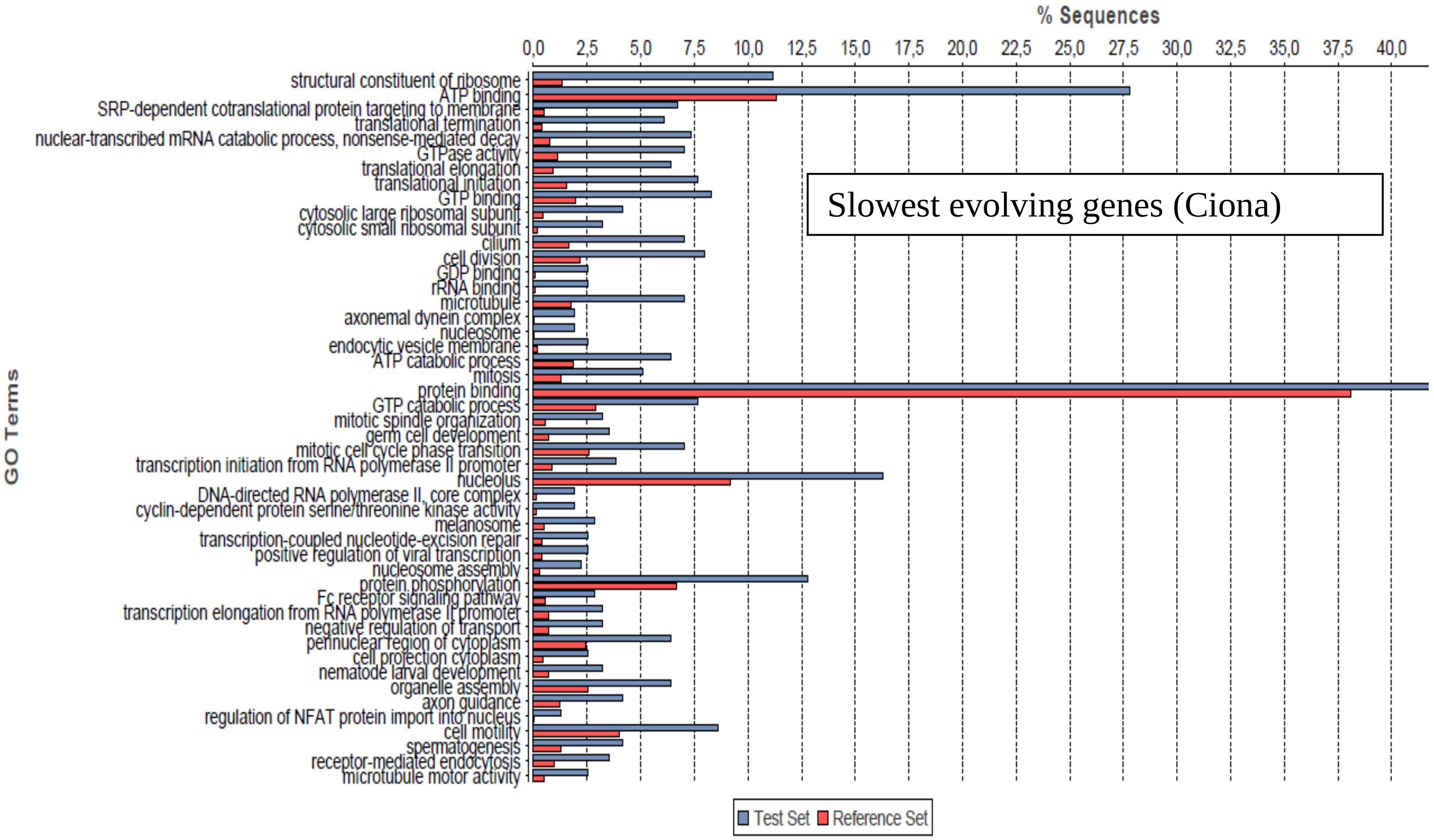

Supplement: Supplementary Data [file supp_evu122_Supplementary_file_2.pdf]
