# Supplementary material for: Evolutionary Genomics of Fast Evolving Tunicates
Source: Genome Biol Evol. 2014 Jul 8;6(7):1724–38. doi: 10.1093/gbe/evu122 (PMC4122922; doi:10.1093/gbe/evu122)
Supplement: Supplementary Data [file supp_evu122_Supplementary_file.docx]

**Table S1 Search of repair genes in Oikopleura**

We searched the translated gene models that are available with the genomes of *Ciona instestinalis*, *C. savygni* and *Oikopleura* *dioica*. For this purpose a HMM profile was built for each one of the genes of interest. These profiles were built in two steps. A first profile was built using only the protein sequences from human, sea urchin and amphioxus *(Homo sapiens* ANNOTATION_RELEASE.104 from NCBI, *Strongylocentrotus purpuratus* version 08/06/12 from NCBI, and [*Branchiostoma floridae*](ftp://ftp.jgi-psf.org/pub/JGI_data/Branchiostoma_floridae/v1.0/Branchiostoma_floridae_v2.0.assembly.fasta.gz) assembly v1.0 from JGI ). These groups of three-way orthologs were identified by reciprocal best hits (using blastp) with the human sequence as initial queries. The annotations associated with these genes (in the respective databases) were used to corroborate orthology assignment.

After aligning each one of these 15 COGs (using clustalw), profiles were constructed with **hmmbuild (HMMER, version 3.1, http:/**hmmer.org**).** The HMM profiles thus obtained were used to search (with hmmsearch) in *C. instestinalis* and *C. savignyi (Ciona intestinalis:* assembly v2.0 from JGI, assembly KH from Genbank (ID GCA_000224145.1, 09/17/2013) and Ensembl version 04/02/14; *Ciona savignyi:* Ensembl version 04/02/14). The best hits (for each one of the genes) in these genomes were checked to be the reciprocal best ones using phmmer (to prevent false positive identification that may correspond to paralogs). If the protein identified this way was the best hit in the human, sea urchin and amphoixus genomes (proteomes), then it is incorporated to the profile (i.e. a new profile is built using the information from this protein from Ciona). Additionally, we cross-checked the Ciona genes’ IDs with the annotation available in other databases [Ensembl, JGI, NCBI, Ghost (<http://ghost.zool.kyoto-u.ac.jp/cgi-bin/gb2/gbrowse/kh/>), Metazome (<http://metazome.net/>), Oma Browser (http://omabrowser.org/cgi-bin/gateway.pl ], Superfamily ( <http://supfam2.cs.bris.ac.uk/SUPERFAMILY/index.html>) and SIMAP (<http://liferay.csb.univie.ac.at/portal/web/simap>) ]

This new profile was used to scan the proteome from Oikopleura (Annotation v1 from Genoscope http://www.genoscope.cns.fr/externe/GenomeBrowser/Oikopleura/), and the positive hits were checked (phmmer) for reciprocity against human, sea urchin, amphioxus and Ciona.

**Gene IDs of the sequences from human, sea urchin and amphioxus**

| **Gene** | ***H. sapiens*** | ***Sea urchin*** | **Amphioxus** |
| --- | --- | --- | --- |
| ID | ID | ID | JGI ID |
| POLB | CAG46601.1 | XP_003729524.1 | jgi\|Brafl1\|123875\|estExt_fgenesh2_pg.C_1310020 |
| APEX2 | AAH02959.1 | XP_784420.3 | jgi\|Brafl1\|277552\|estExt_gwp.C_370032 |
| LIG3 | NP_002302.2 | XP_786357.3 | jgi\|Brafl1\|119901\|estExt_fgenesh2_pg.C_360100 |
| ATM | NP_000042.3 | XP_003729026.1 | jgi\|Brafl1\|85377\|fgenesh2_pg.scaffold_141000026 |
| CHEK2 | NP_009125.1 | XP_794585.3 | jgi\|Brafl1\|230456\|e_gw.272.90.1 |
| NBN | NP_002476.2 | XP_783339.3 | jgi\|Brafl1\|121621\|estExt_fgenesh2_pg.C_690076 |
| RAD52 | NP_602296.2 | XP_784279.3 | jgi\|Brafl1\|94088\|fgenesh2_pg.scaffold_247000015 |
| XRXX5 | NP_066964.1 | XP_788472.3 | jgi\|Brafl1\|164247\|gw.1.181.1 |
| XRCC6 | NP_001460.1 | XP_001180453.1 | jgi\|Brafl1\|281658\|estExt_gwp.C_1100146 |
| LIG4 | NP_001091738.1 | XP_787257.3 | jgi\|Brafl1\|282874\|estExt_gwp.C_2760092 |
| XRCC4 | NP_003392.1 | XP_001199052.2 | jgi\|Brafl1\|130664\|estExt_fgenesh2_pg.C_4520030 |
| NHEJ1 | NP_079058.1 | XP_001187188.2 | jgi\|Brafl1\|131400\|estExt_fgenesh2_pg.C_5320026 |
| DNA-PKc | NP_001075109.1 | XP_781813.3 | jgi\|Brafl1\|85202\|fgenesh2_pg.scaffold_139000049 |
| DCLRE1C | NP_001029027.1 | XP_781856.3 | jgi\|Brafl1\|215154\|e_gw.61.284.1 |
| APTX | NP_001182179.1 | XP_797394.3 | jgi\|Brafl1\|252705\|e_gw.628.4.1 |
| MSH3 | AAB47281.1 | XP_001193092.2 | jgi\|Brafl1\|133439\|estExt_fgenesh2_pg.C_16070001 |

**Gene IDs of tunicate sequences**

| **Gene** | ***Ciona instestinalis*** | ***Ciona savygni*** | ***Oikopleura dioca*** |
| --- | --- | --- | --- |
| ID | JGI ID | Ensembl ID | Genoscope ID |
| POLB | jgi\|Cioin2\|219811\|fgenesh3_pg.C_chr_09p000020 | ----- | SNAPOd2T00021809001 |
| APEX2 | ----- | ENSCSAVP00000010265 | -- |
| LIG3 | jgi\|Cioin2\|260784\|gw1.10p.138.1 | ENSCSAVP00000011468 | -- |
| ATM | jgi\|Cioin2\|274957\|gw1.12q.732.1 | ENSCSAVP00000006863 | -- |
| CHEK2 | jgi\|Cioin2\|287385\|estExt_fgenesh3_pg.C_chr_02q1714 | ENSCSAVP00000008401 | -- |
| NBN | ----- | ----- | SNAPOd2T00015064001 |
| RAD52 | ----- | ----- | -- |
| XRXX5 | jgi\|Cioin2\|270692\|gw1.215.15.1 | ENSCSAVP00000000283 | -- |
| XRCC6 | jgi\|Cioin2\|280036\|gw1.13q.456.1 | ENSCSAVP00000001703 | -- |
| LIG4 | jgi\|Cioin2\|260123\|gw1.03q.407.1 | ENSCSAVP00000017544 | -- |
| XRCC4 | jgi\|Cioin2\|287449\|estExt_fgenesh3_pg.C_chr_03p0091 | ----- | -- |
| NHEJ1 | jgi\|Cioin2\|295297\|estExt_fgenesh3_pg.C_920013 | ----- | -- |
| DNA-PKc | jgi\|Cioin2\|261176\|gw1.03q.462.1 | ENSCSAVP00000019700 | -- |
| DCLRE1C | jgi\|Cioin2\|263187\|gw1.92.32.1 | ----- | -- |
| APTX | jgi\|Cioin2\|297959\|estExt_fgenesh3_kg.C_chr_07q0165 | ENSCSAVP00000013710 | -- |
| MSH3 | ----- | ----- | -- |

**Figures S1, S2 and S3. Evolutionary rates in most accelerated *Oikopleura* and *Ciona* genes.**

**Figures S1** In the figure that is presented below, we plot “acceleration” (Oikoploeura/vertebrates) versus the basal evolutionary rate in vertebrates (panel A) and also Amphioxus (panel B). As it can be observed, the most “accelerated” genes from Oikopleura, are not particularly fast evolvers neither in amphioxus nor in vertebrates. In Oikopleura (panel C), the rates of highly accelerated genes range from slow to fast.**Figures S1. Continuation Figure S2** Histograms showing the distance distribution between present day vertebrate genes (represented *by Bos taurus*) and their common ancestor with tunicates (branch c) in very accelerated *Oikopleura* genes ((a+D1)/c>5) and rest of the genome (A). In panel B, a similar histograms showing the distance distribution for *Amphioxus* and its common ancestors with vertebrates (branch ***d***), in very accelerated genes and rest of genome

Rate in Vertebrates (branch ***c***)

Rate in Amphioxus (branch ***d***)

(a+D1)/c

(***a***+D1)/c ratios versus rate in Amphioxus (branch ***d***). Red points most accelerated genes (***a***+D1)/c>5

B

(a+D1)/c

(***a***+D1)/c ratios versus rate in vertebrates (branch ***c***). Red points most accelerated genes (***a***+D1)/c>5

A

(a+D1)/c

(***a***+*D1*)/c ratios versus rate in Oikopluera (branch ***a***). Red points most accelerated genes (***a***+D1)/c>5

C

Rate in Oikopleura (branch ***a***)


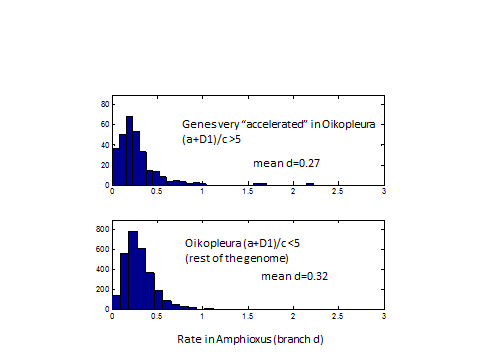


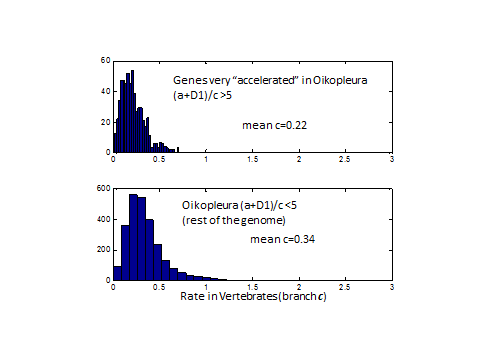


B

A

**Figure S3 Evolutionary rates in most accelerated *Ciona* genes.**

In panel A, we plot “acceleration” (Ciona/vertebrates) versus the basal evolutionary rate in vertebrates (branch c). Panel B (Ciona/vertebrates) versus rate in Ciona (branch b).

“Acceleration” in *Ciona*. In red most accelerated genes (b+D1)/c>2.55

(***b+D1***)/***c***

A

B

Rate in Vertebrates (branch ***c***)

In red most accelerated genes (b+D1)/c>2.55

**Figure S4. GO enrichment analysis in least and non-accelerated genes.**

(***b+D1***)/***c***

Rate in *Ciona* (branch ***b***)


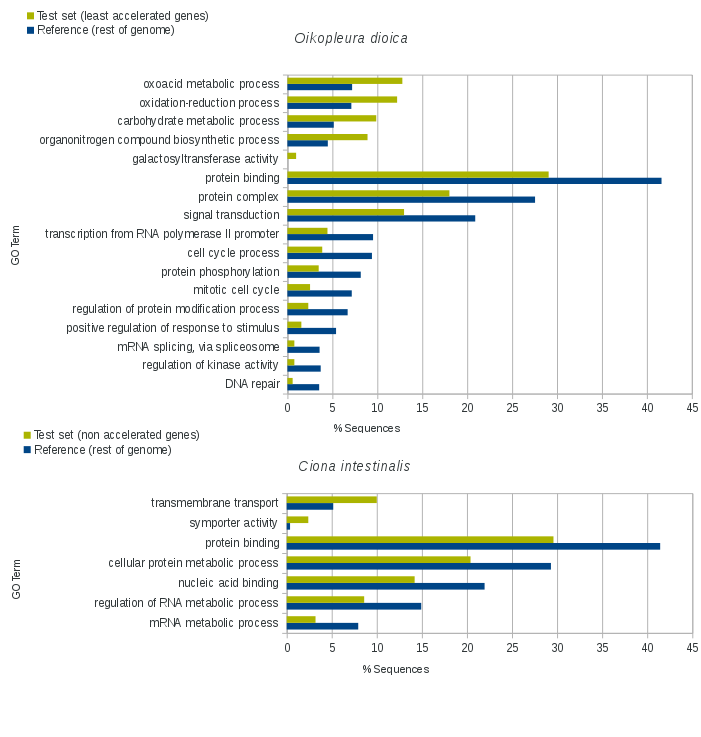


*Oikopleura* (a+D1)/c <1.8

*Ciona* (b+D1)/c ≤1.1
